# Signaling through hepatocyte vasopressin receptor 1 protects mouse liver from ischemia-reperfusion injury

**DOI:** 10.18632/oncotarget.12472

**Published:** 2016-10-04

**Authors:** Xiqiang Liu, Gaojian Luo, Jingbo Jiang, Tonghui Ma, Xiaozhu Lin, Liping Jiang, Jilin Cheng, Ran Tao

**Affiliations:** ^1^ Department of Hepatobiliary-Pancreatic Surgery, Zhejiang Provincial People's Hospital (ZJPPH), Hangzhou, Zhejiang, PR China; ^2^ Department of General Surgery, Affiliated Yiwu Hospital, Wenzhou Medical University, Yiwu, Zhejiang, PR China; ^3^ Department of Surgery, Qilu Hospital, Shandong University School of Medicine, Jinan, Shandong, PR China; ^4^ Department of Radiology, Ruijin Hospital, Shanghai Jiaotong University School of Medicine, Shanghai, PR China; ^5^ Department of Gastroenterology, Shanghai Public Health Clinical Center, Fudan University, Shanghai, PR China

**Keywords:** apoptosis, hepatocyte, hypoxia, inflammation, vasopressin receptor, Pathology Section

## Abstract

Terlipressin has been used extensively in the management of certain complications associated with end-stage liver diseases (ESLDs). In our pilot study, terlipressin treatment showed beneficial effects on liver function in patients with decompensated cirrhosis, however whether it plays a role in liver ischemia-reperfusion injury (IRI) remains unknown. Using a mouse nonlethal hepatic IR model, we found terlipressin administration significantly ameliorated IR-induced liver apoptosis, necrosis and inflammation. Furthermore, despite its known effect on visceral vasoconstriction, hemodynamic evaluation of murine hepatic tissue after IR revealed no change of overall hepatic blood flow after terlipressin treatment. Further studies identified the upregulation of vasopressin receptor 1 (V1R) expression on hepatocytes upon IR. In isolated hepatocyte hypoxia/reoxygenation model, the active component of terlipressin, lysine vasopressin, conferred hepatocytes resistant to oxidative stress-induced apoptosis. Mechanistic studies revealed the V1R engagement activated the Wnt/β-catenin/FoxO3a/AKT pathway, which subsequently circumvented the proapoptotic events, thus ameliorated hepatocyte apoptosis. Furthermore, genetic knockdown of V1R expression in hepatocyte cell lines or blockade of this signaling pathway abrogated such protective effect. Conclusion: These data highlight the functional importance of the hepatocyte V1R/Wnt/β-catenin/FoxO3a/AKT pathway in protecting liver from oxidative stress-induced injury.

## INTRODUCTION

Terlipressin (3′glycyl lysine vasopressin) is a well-recognized rising star in the medical management of advanced liver cirrhosis and portal hypertension, and has been proven to be the only drug so far which can improve the survival of such patients [1]. As a synthetic vasopressin analogue, terlipressin has minimal activity per se, however the active form lysine vasopressin can be slowly released when the glycyl residues on the N-terminal are removed by aminopeptidase in the body, resulting in a significantly extended biological half-life (4~6 hours) in comparison to the physiological vasopressin. There are two major forms of vasopressin receptors in the human body, namely V1 and V2 receptors (V1R and V2R). V1R is mainly expressed by vascular smooth muscle cells, but can also be detected in the liver and other organs; while V2R is predominantly distributed in the renal collecting duct epithelial cells [2]. Terlipressin has a selectively high affinity for V1R, while its anti-diuretic activity stimulated by V2R is only 3% of that in the natural vasopressin. Terlipressin can selectively contract the gastrointestinal capillary bed and dramatically reduce portal pressure, thus it is mainly used for the treatment of esophageal variceal bleeding. Recent clinical trials also reveal terlipressin can achieve good outcomes in the management of other common co-morbidities of advanced portal hypertension such as hepatorenal syndrome, refractory ascites and pleural effusion, making it an ideal drug for integrated treatment of advanced liver cirrhosis and its complications [3].

Besides its dominant expression in vascular smooth muscle cells, V1R has been found in several other tissues or organs, including the liver, blood platelets, vascular endothelial cells, kidney, spleen, adrenal cortex, brain, adipocytes and the reproductive organs,etc. Although the expression of vasopressin receptor on hepatocytes has been discovered long time ago [4], most studies have focused on the G protein-coupled receptor (GPCR)-stimulated calcium signaling in hepatocytes [2, 4], while the biological significance of V1R has rarely been demonstrated *in vivo*. Russell et al. previously found that liver DNA synthesis induced by partial hepatectomy was impaired in genetically AVP-deficient rats [5]. Nicou et al. also showed that vasopressin contributed to growth initiation and bile flow stimulation in the early stages of liver regeneration [6]. IRI is a common cause of liver damage occurring during hepatic resection or transplantation. However, no data has been presented so far regarding the role of vasopressin on hepatic function in a clinically relevant liver IR model. This can be largely attributed to the short half-life of natural vasopressin, which requires continuous infusion and makes animal study difficult. The introduction of the synthetic vasopressin analogue terlipressin provides a feasible means to test the biological effect of vasopressin *in vivo*.

Because terlipressin reduces portal pressure by directly or indirectly causing splanchnic and peripheral vasoconstriction, a major concern has been raised regarding whether the use of terlipressin can worsen the already compromised liver function. To address this issue, we first reviewed the medical records from a group of patients with ESLD who received terlipressin treatment. To our surprise, terlipressin treatment didn't show any detrimental effect on hepatic function, instead it significantly improved the biochemical parameters of hepatocellular injury. Given the paucity of data regarding the effect of terlipressin on hepatic IR, we therefore exploited a mouse liver warm IR model to evaluate the effect of terlipressin on hepatic function, and an *in vitro* hepatocyte hypoxia/reoxygenation (HR) model to clarify the underlying mechanism.

## RESULTS

### Terlipressin treatment improves liver function in patients with ESLD

**Figure 1:**
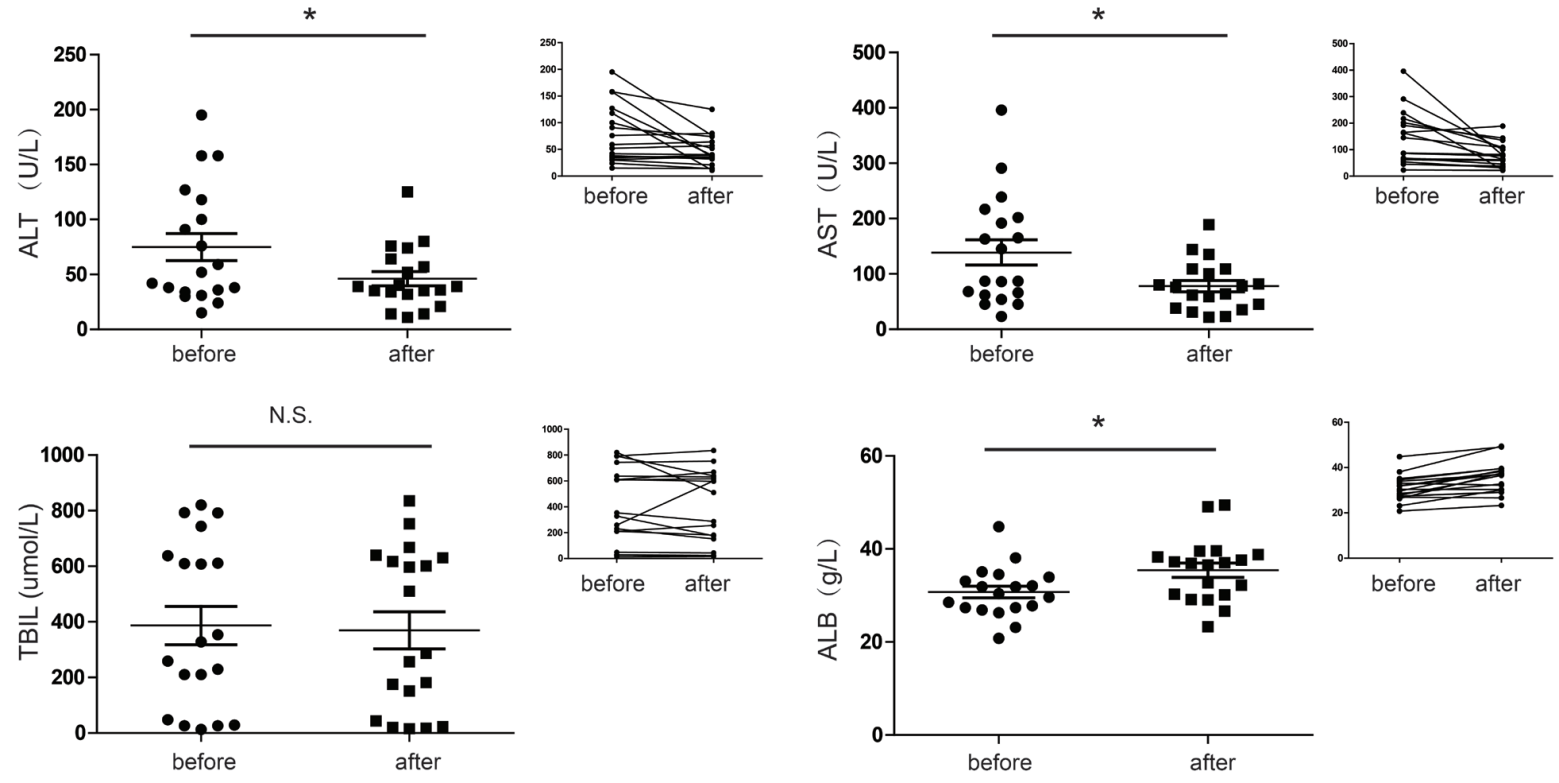
Changes of liver function (mean ±SD) before and after terlipressin treatment in ESLD patients Enrolled patients with ESLD were treated with terlipressin (1-2 mg/day) for 3-7 days. Individual values of serum ALT, AST, TBIL and ALB were measured. *p < 0.05. ALT, alanine transaminase; AST, aspartate aminotransferase; TBIL, total bilirubin; ALB, albumin.

During the study period, nineteen patients underwent terlipressin treatment for various causes. The demographic characteristics of the study group were shown in Supplementary Table 1. Patients included had moderate-severe liver failure, as indicated by markedly impaired liver function tests and high Child-Pugh-Turcotte scores (CPT scores). In the whole cohort of patients, there was a marked reduction in alanine transaminase (ALT) and aspartate aminotransferase (AST), as well as significantly increased serum albumin (ALB) level after treatment with terlipressin, indicating lessened hepatocyte injury and improved hepatocyte synthetic function. Quite surprisingly, despite its cholerectic effect in mouse bile duct ligation models (Liu X, Tao R, manuscript in preparation), no significant change was observed in serum bilirubin level after terlipressin treatment. (Figure 1). There was also a notable reduction in CPT score (9.95±1.22 before treatment vs. 8.58±1.47 after treatment, *p* < 0.01) after treatment with terlipressin. These data led us to pursue further research regarding the effect of terlipressin on liver injury as well as the underlying mechanism.

### Terlipressin doesn't alter the overall mouse hepatic blood perfusion *in vivo*

**Figure 2:**
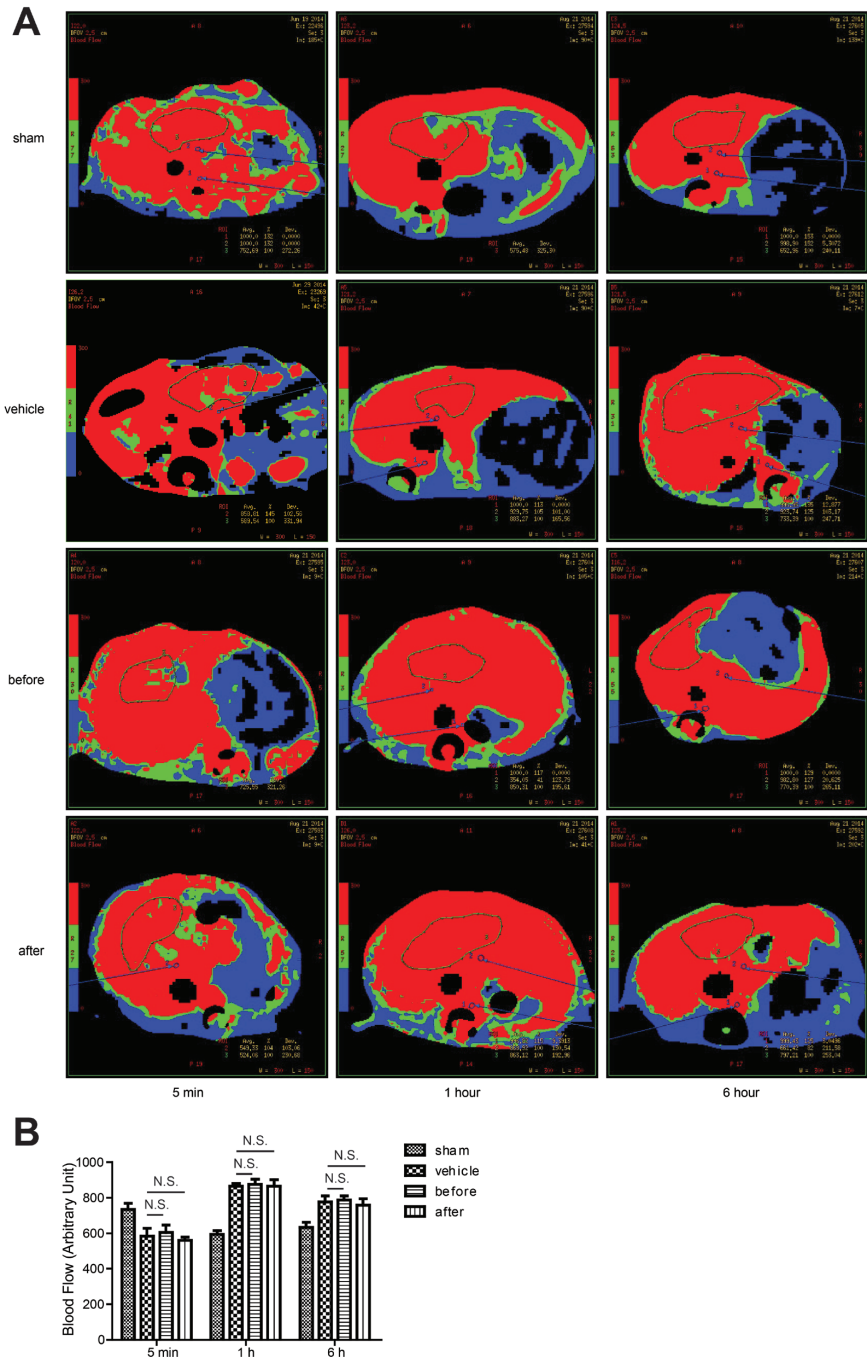
The effect of Terlipressin on the overall mouse hepatic blood perfusion *in vivo*. B6 mice were either sham-operated or subjected to 90 minutes of partial warm ischemia, followed by 5 min, 1 hour or 6 hours of reperfusion. Mice in groups treated with terlipressin before ischemia (before group, 0.1mg/kg iv) or after initiation of reperfusion (after group, 0.1mg/kg iv) as well as mice in vehicle groups pretreated with the same volume of normal saline were subjected to ischemia (*n* = 3-6/group). Liver blood flow was analyzed by computed tomography. **A.** Representative CT images from at least three mice for each time point were shown and **B.**. NS, no significance.

Given that terlipressin is a known vasoconstrictor which reduces hepatic portal inflow [7], we first determined whether such hepatoprotective effect was caused by reduced hepatic tissue perfusion in the IR lobes. However, CT evaluation showed no significant difference of the overall blood perfusion in the IR tissues between terlipressin and vehicle treated groups at different time points (Figure 2A and 2B). This unexpected finding led to the hypothesis that V1R expression on hepatocytes might play a critical role in the regulation of hepaocyte function.

### Terlipressin treatment dramatically attenuates hepatic injury and inflammation

**Figure 3:**
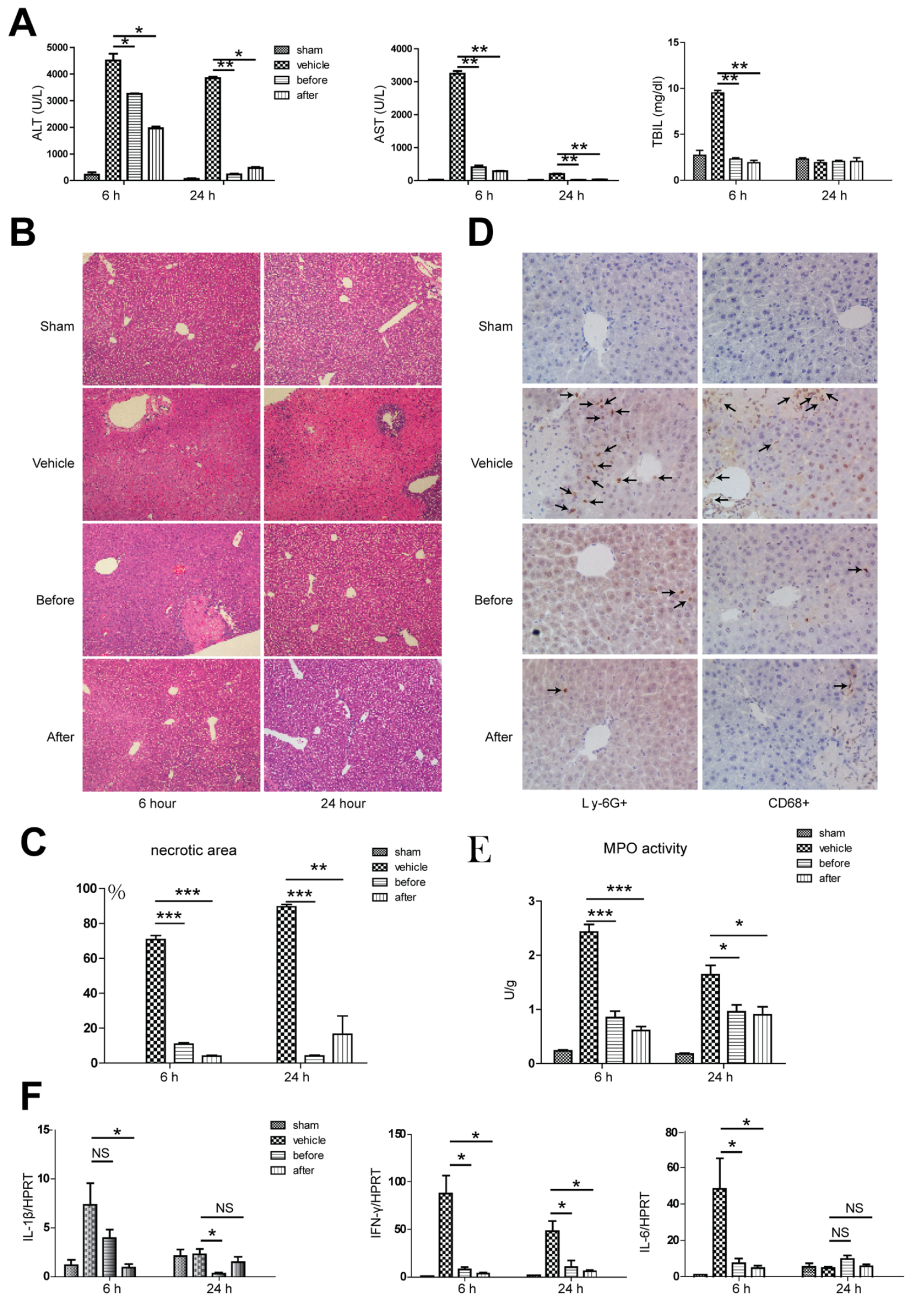
Terlipressin treatment ameliorates hepatic injury and inflammation B6 mice were either sham operated (sham group) or subjected to 90 minutes of partial warm ischemia, followed by 6 or 24 hours of reperfusion. Those subjected to ischemia (*n* = 3-6/group) were either treated with terlipressin before ischemia (before group, 0.1mg/kg iv) or right after initiation of reperfusion (after group, 0.1mg/kg iv), while mice in the vehicle control group were treated with the same volume of normal saline. Serum and liver samples were harvested at 6 or 24 hours after reperfusion. Liver damage was analyzed by **A.** serum ALT, AST, TBIL levels and **B.** and **C.** liver histology (representative H&E staining; 100 × magnification). Accumulation of neutrophils and macrophages in IR livers after administration of terlipressin was demonstrated by **D.** Ly-6G+ neutrophils, CD68+ macrophages (400×magnification) and **E.** MPO levels. **F.** qRT-PCR detection of levels. Compared to the vehicle group, *p < 0.05, **p < 0.01, ***p < 0.001. ALT, alanine transaminase; AST, aspartate aminotransferase; TBIL, total bilirubin; IR, ischemia-reperfusion.

We used a well-established mouse nonlethal partial hepatic IR model to test the possible protective effect of terlipressin against liver injury. At 6 and 24 hours post-reperfusion, terlipressin treatment either before or after reperfusion dramatically ameliorated hepatic IRI, manifested as decreased ALT and AST levels at both time points in comparison to mice treated with vehicles. Total bilirubin levels only decreased after 6 hours of reperfusion with terlipressin treatment (Figure 3A). Pathological examination revealed better preserved lobular structure and significantly less necrosis (Figure 3B and 3C), as well as less neutrophil and macrophage infiltration by immunohistochemical staining after terlipressin treatment in comparison to vehicle-treated mice at 6 hours post-reperfusion (Figure 3D). In line with the pathological findings, we detected decreased MPO activity in the hepatic tissue after receiving terlipressin treatment (Figure 3E). Next, given hepatic IRI is essentially an inflammatory response, we attempted to examine the cytokine milieu in the hepatic tissue. Compared to the vehicle group, real-time quantitative PCR assay showed that at 6 hours post-reperfusion, terlipressin treatment either before or after ischemia significantly decreased the gene expression of interferon-γ (IFN-γ) and interleukin-6 (IL-6), while only post- but not pre-ischemia terlipression therapy showed decreased interleukin-1β (IL-1β) expression. We also detected a different intrahepatic gene expression profile at 24 hours after reperfusion. Terlipressin treatment either before or after ischemia significantly decreased the gene expression of IFN-γ, while only pre- but not post-ischemia terlipression therapy showed decreased IL-1β, and no change of gene expression was identified for IL-6 (Figure 3F).

### Terlipressin significantly ameliorates apoptosis in mouse livers undergone IR

**Figure 4:**
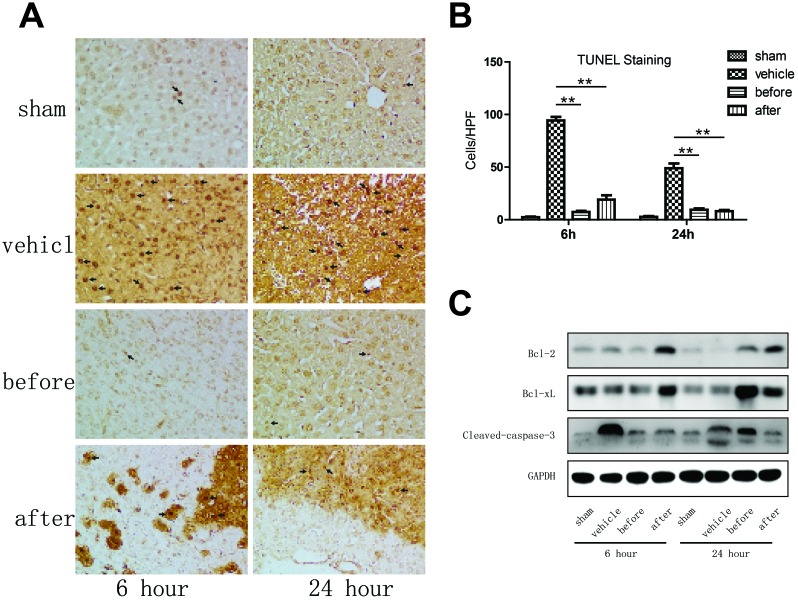
Terlipressin markedly ameliorates apoptosis in mouse livers undergone IRI **A.-C.** Liver samples were harvested from B6 mice that were either sham-operated or subjected to 90 minutes of partial warm ischemia, followed by 6 or 24 hours of reperfusion. Those subjected to ischemia (*n* = 3-6/group) were either treated with terlipressin before ischemia (before group, 0.1mg/kg iv) or right after initiation of reperfusion (after group, 0.1mg/kg iv), while mice in the vehicle control group were treated with the same volume of normal saline. **A.** Detection of apoptotic cells (dark arrows) within the IR hepatic lobes using the TUNEL staining (400×magnification). **B.** Quantification of TUNEL positive cells in IR hepatic lobes with or without terlipressin treatment. TUNEL positive cells were calculated within 15 randomly selected fields and were expressed as mean±SD. **p < 0.01 as compared with vehicle group. **C.** Western blot analysis of BCL-2, BCL-XL and cleaved-caspase-3 in IR liver lobes. Representative blots from at least three mice for each time point were shown, and densitometric values were shown in supplementary figure 1 (Figure S1). IR, ischemia-reperfusion; TUNEL, terminal deoxynucleotidyl transferase-mediated dUTP nick end labeling; BCL-2, B-cell lymphoma 2; BCL-XL, B-cell lymphoma-extra large; ALT, alanine transaminase; AST, aspartate aminotransferase.

Terlipressin treatment diminished otherwise abundant hepatocellular necrosis/apoptosis, manifested as reduced frequency of TUNEL positive cells within the liver (Figure 4A and 4B). Western blot analysis showed that at 6 hours after reperfusion, only post-reperfusion but not pre-ischemia terlipression therapy increased the protein expression of BCL-2 and BCL-XL, while terlipressin treatment both before and after reperfusion significantly decreased the expression of cleaved-caspase-3, compared to the vehicle group. The expression of BCL-2 and BCL-XL remained higher after 24 hours of reperfusion with terlipressin treatment than the vehicle group. Only post-reperfusion but not pre-ischemia terlipressin therapy reduced liver cleaved-caspase-3 expression after 24 hours of reperfusion (Figure 4C and S1).

### Increased expression of V1R on hepatocytes upon IR

**Figure 5:**
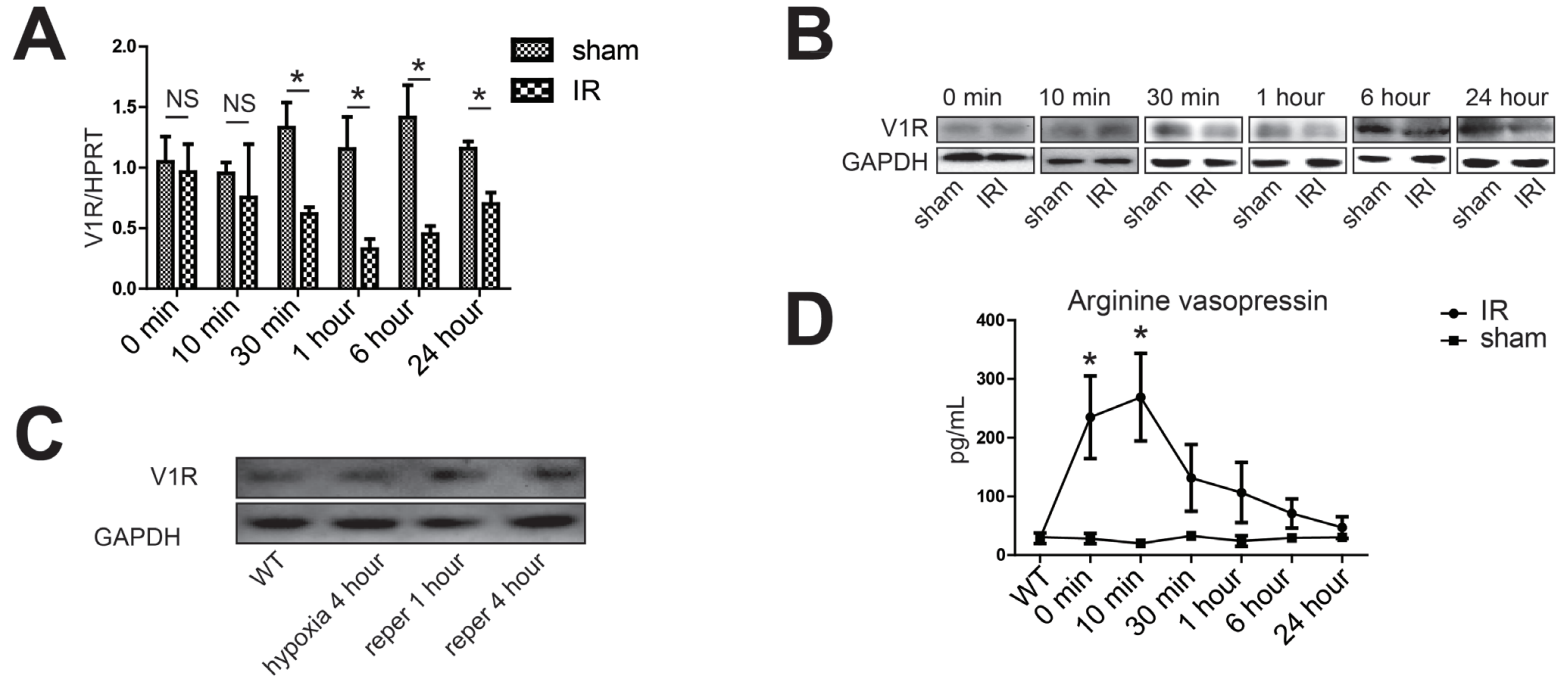
Activation of the vasopressin system during liver IR **A.**, **B.**, **D.** Liver samples were harvested from B6 mice that were either sham-operated (sham group) or subjected to 90 minutes of partial warm ischemia, followed by 0, 10, 30 minutes, 1, 6 or 24 hours of reperfusion (IR group, *n* = 3-6/group). **A.** The V1R gene expression was detected by real-time quantitative PCR, data were expressed as fold increase (mean±SD) above the sham group after 0 minute of reperfusion (set as 1). **B.** Western blot analysis of V1R expression in IR liver lobes. Densitometric values were shown in Figure S2A. **C.** Western blot analysis of V1R expression in isolated hepatocytes. Primary hepatocytes were subjected to hypoxia (SO_2_:3%) for 4 hours, hypoxia for 4 hours and restoration of oxygen supply (95% O_2_/5% CO_2_) for 1 (reper 1 hour) or 4 (reper 4 hour) hours. Primary hepatocytes from wild type B6 mice (WT) were subjected to normal oxygen supply (21% O_2_/5% CO_2_). Densitometric values of the western blot result were shown in Figure S2B. **D.** Serum concentrations of arginine vasopressin during hepatic IR. Data were expressed as mean±SD, *p < 0.05 as compared with sham group, NS, no significance. V1R, arginine vasopressin V1 receptor; PCR, polymerase chain reaction; IR, ischemia-reperfusion.

Given that V1R is the only receptor for terlipressin in the liver, we next determined serial expression of V1R within the IR lobes as well as the serum level of natural vasopressin secreted by the pituitary gland after hepatic IR. Compared with sham controls, the overall V1R messenger RNA and protein levels in the IR lobetic tissues didn't change upon ischemia but rather decreased after 30 minutes of reperfusion (Figure 5A, 5B and S2A). However, the protein expression of V1R was significantly increased on isolated hepatocytes in an *in vitro* hypoxia/reoxygenation system mimicry of the liver IRI model *in vivo* (Figure 5C and S2B), suggesting the overall decrease of V1R expression in IR tissues can be largely attributed to the dampened expression of VIR in vascular smooth muscle cells within the liver. On the other hand, serum concentration of endogenous arginine vasopressin (AVP) increased progressively after the 90-min ischemic insult, peaking at 10 minutes after reperfusion and decreased thereafter (Figure 5D). Such cell-specific and dynamic pattern of V1R and vasopressin change further supported the rational to focus on the role of hepatocyte V1R signaling in ameliorating liver IRI.

### Lysine vasopressin protects hepatocytes from hypoxia/reoxygenation-induced injury in a V1R-dependent manner

**Figure 6:**
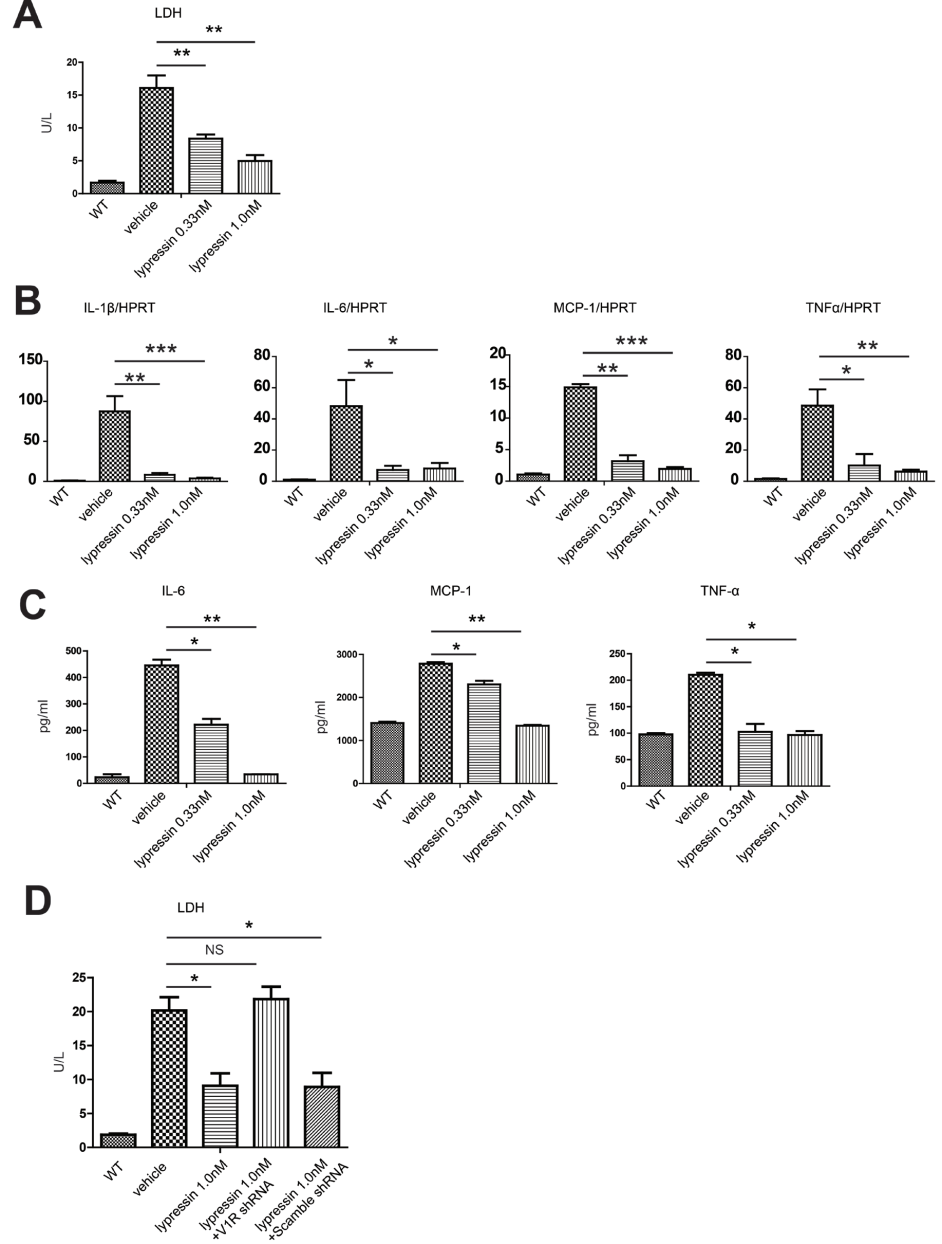
Lysine vasopressin protects primary hepatocytes from hypoxia/reoxygenation-induced injury *in vitro* Primary mouse hepatocytes were subjected to hypoxia for 4 hours (3% O_2_) and restoration of oxygen supply (95% O_2_/5% CO_2_) for 4 hours in the presence or absence of lysine vasopressin. WT control represented primary hepatocytes that were subjected to normal oxygen supply (21% O_2_/5% CO_2_). **A.** LDH level in the supernatant of cultured hepatocytes. *p < 0.05, **p < 0.01 as compared to the vehicle group. **B.** qRT-PCR detection of IL-1β, IL-6, MCP-1 and TNF-α in hepatocytes. Data were normalized to HPRT gene expression and expressed as fold increase above the WT control group (set as 1). **C.** ELISA detection of IL-6, MCP-1 and TNF-α in the supernatant. WT, wild type; LDH, lactate dehydrogenase; BCL-2, B-cell lymphoma 2; BCL-XL, B-cell lymphoma-extra large. **D.** Lentiviral-mediated V1R shRNA transfection inhibited hypoxia/reoxygenation-induced injury in NCTC cells.

To further confirm the functional importance of V1R expression on isolated hepatocytes, we used an *in vitro* hypoxia/reoxygenation system. As expected, treatment of hepatocytes with 4 hours of hypoxia and restoration of oxygen supply for another 4 hours resulted in increased release of lactate dehydrogenase (LDH) into the supernatant. The active form of terlipressin, lysine vasopressin, markedly decreased hepatocyte damage evidenced by reduced LDH levels (Figure 6A). In comparison to the vehicle group, lysine vasopressin also significantly decreased hypoxia/reoxygenation-induced mRNA expression (Figure 6B) as well as cytokine release (Figure 6C) of several inflammatory mediators such as IL-1β, IL-6, MCP-1 and TNFα.

To exclude the possibility that vasopressin may act through other pathways independent of its receptor, V1R was knocked-down by short-hairpin RNA (shRNA) in an immortal mouse hepatocyte cell line, NCTC cells. Similarly, lysine vasopressin markedly decreased NCTC cell damage evidenced by reduced LDH levels after 4 hours of hypoxia and restoration of oxygen supply for another 4 hours. However, silencing V1R by shRNA readily abrogated the hepatoprotective effect of vasopressin (Figure 6D), suggestive of an irreplaceable role of the vasopressin-V1R system in ameliorating liver IR.

### Ligation of V1R inhibits the Wnt/β-catenin/ FoxO3a/ AKT pathway to exert its hepatoprotective effects

**Figure 7:**
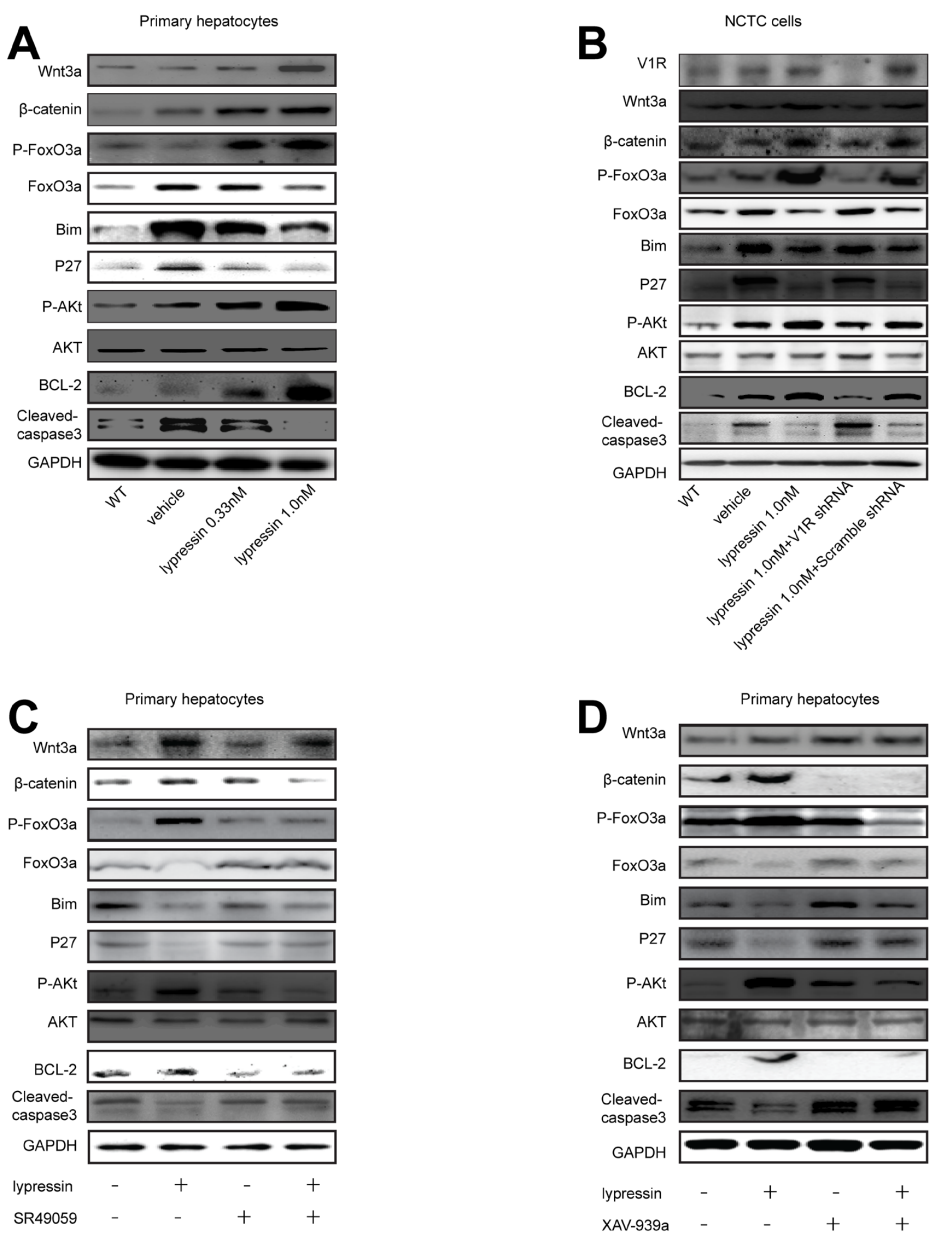
Lysine vasopressin exerts its hepatoprotective effects *via* V1R-mediated activation of the Wnt/β-catenin /FoxO3a/AKT pathway Primary hepatocytes or transfected NCTC cells were subjected to hypoxia (SO_2_:3%) for 4 hours and restoration of oxygen supply (95% O_2_/5% CO_2_) for 4 hours in the presence or absence of lysine vasopressin. **A.** Western blotting analysis of Wnt3a, β-catenin, Phospho-FoxO3a, FoxO3a, Bim, P27, Phospho-AKT, AKT, BCL-2 and cleaved-caspase-3 in hepatocytes, and densitometric values were shown in Figure S3. **B.** NCTC cells were transfected with control or V1R lentiviral shRNA and then treated with lysine vasopressin. Western blotting analysis of V1R, Wnt3a, β-catenin, Phospho-FoxO3a, FoxO3a, Bim, P27, Phospho-AKT, AKT, BCL-2 and cleaved-caspase-3 in cells, and densitometric values were shown in Figure S4. **C.**, **D.** Primary hepatocytes were treated with vehicle or **C.** SR49059 (10 uM) for 10 minutes or **D.** XAV939a (1uM) for 24 hours and then subjected to hypoxia (SO_2_:3%) for 4 hours and restoration of oxygen supply (95% O_2_/5% CO_2_) for 4 hours in the presence or absence of lysine vasopressin. Western blot analysis of Wnt3a, β-catenin, Phospho-FoxO3a, FoxO3a, Bim, P27, Phospho-AKT, AKT, BCL-2 and cleaved-caspase-3 in hepatocytes, and densitometric values were shown in Figures S5 and S6, respectively. V1R, arginine vasopressin receptor 1; FoxO3a, forkhead box O 3a; BCL-2, B-cell lymphoma 2.

As reported previously, the Wnt/β-catenin/FoxO signaling played an essential role in the hepatoprotection against oxidative stress-induced cell death through cross-talk with AKT [8]. This prompted us to investigate the possible relationship between V1R and the Wnt/β-catenin/FoxO/AKT signaling in mediating the beneficial effect of vasopressin against hypoxic injury. Primary hepatocytes underwent 4 hours of hypoxia and restoration of oxygen supply for 4 hours in the presence of 0.33 or 1.0 nM lysine vasopressin. Our data showed that lysine vasopressin treatment ameliorated hepatocyte apoptosis as shown by decreased caspase-3 cleavage and increased anti-apoptotic BCL-2 expression in a dose-dependent manner. Meanwhile, Wnt3a, β-catenin, phospho-AKT and phospho-FoxO3a proteins were markedly increased, while FoxO3a protein was markedly decreased after lysine vasopressin treatment in a dose-dependent manner. Furthermore, Bim and P27, two known FoxO3a targets which directly induce apoptosis, were decreased after lysine vasopressin treatment (Figure 7A and S3).

To further confirm the critical role of V1R-Wnt/β-catenin/FoxO/AKT signaling in the hepatoprotective effect of vasopressin, we took advantage of both genetic approach and small molecule inhibitors. First, V1R expression in NCTC cells was knocked down using shRNA. NCTC cells were treated with 4 hours of hypoxia and 4 hours of restoration of oxygen supply in the presence or absence of 1.0 nM lysine vasopressin. shRNA treatment decreased V1R protein expression compared with the control shRNA. Knock-down of V1R prevented the anti-apoptotic effects of lysine vasopressin as shown by increased caspase-3 cleavage and decreased BCL-2 expression. Furthermore, knockdown of V1R diminished the expression of Wnt3a, β-catenin, phospho-AKT and phospho-FoxO3a, while upregulated total FoxO3a, Bim and P27 (Figure 7B and S4). These results indicated that lysine vasopressin may protect hepatocytes from oxidative stress-induced apoptosis via V1R-mediated Wnt/β-catenin/FoxO3a/AKT pathway. Next, we used specific small molecule inhibitor to block V1R in primary hepatocytes. The vasopressin/V1R-stimulated upregulation of Wnt3a, β-catenin, phospho-FoxO3a and phospho-AKT as well as the downregulation of FoxO3a, Bim and P27 were readily reversed by the V1R antagonist SR49059. Similarly, the anti-apoptotic effect of lysine vasopressin was blocked by SR49059, evidenced by substantial downregulation of BCL-2 and upregulation of caspase-3 cleavage (Figure 7C and S5). We also used small molecule inhibitors to block the key node of the above-mentioned signaling pathway in primary hepatocytes. Consistently, the vasopressin's hepatoprotective effect against hypoxia/reoxygenation-induced injury was readily reversed by incubation with the Wnt signaling inhibitor XAV-939a, with substantial downregulation of BCL-2 and upregulation of caspase-3 cleavage. Meanwhile, the lysine vasopressin-stimulated upregulation of β-catenin, phospho-FoxO3a and phospho-AKT and downregulation of FoxO3a, Bim and P27 were reversed by XAV-939a (Figure 7D and S6.)

## DISCUSSION

**Figure 8:**
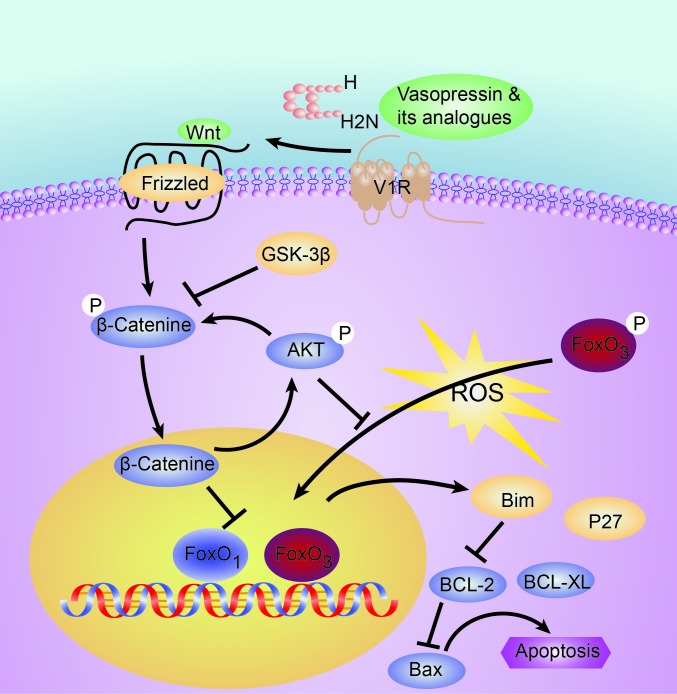
Schematic illustration of the tentative signaling pathways activated by V1R ligation which may protect the liver from redox-induced apoptosis V1R, arginine vasopressin V1 receptor; FoxO3a, forkhead box O 3a.

Liver IRI is a common pathological process in liver surgery and transplantation, which has considerable impact on the patient outcome after surgery. We found the vasopressin analogue terlipressin treatment effectively ameliorated the hepatocyte injury after warm IR in mice via hepatocyte V1R-mediated activation of the Wnt/β-catenin/FoxO3a/AKT pathway, which subsequently obscured the proapoptotic events driven by FoxO3-activated transcription of Bim and P27. Although it was previously shown that vasopressin stimulated phospholipase C and the mitogen-activated protein kinases (MAPKs), extracellular signal-induced kinases 1/2 (ERK1/2) and p38 to exert downstream effect [9–13], ours represents the first study to reveal the mechanism of vasopressin analogue on IR-induced hepatocyte apoptosis via the Wnt/β-catenin/FoxO3a/AKT pathway in mice (Figure 8). We further identified that such hepatoprotective effect was unlikely caused by decreased portal blood flow, a well-known hemodynamic effect of terlipressin, since the overall perfusion of the hepatic tissue remained unchanged, most likely due to the “buffer effect” of hepatic artery. Interestingly, we found in the *in vivo* model that although administration of terlipressin at both time points were highly effective against hepatic IRI given the extended half-life of this synthetic peptide, post-reperfusion treatment seemed to be superior to the pre-ischemia therapy as evidenced by the better preserved liver structure, less necrosis and inflammation, better liver function test as well as increased expression of anti-apoptotic proteins (Figures 3 and 4). This could be mostly attributed to the dramatically increased V1R expression after reperfusion to ensure better ligand-receptor engagement. Finally, the *in vitro* hypoxia/re-oxygenation results showed a dose-dependent hepatocyte protective effect against oxidative stress-induced injury after lysine vasopressin treatment. Collectively, these data support a direct hepatoprotective role of vasopressin via hepatocyte V1R ligation.

The anti-apoptotic effect of vasopressin has only been briefly documented in previous studies in renal collecting duct via V2R [14] and neurons via V1R [15], respectively, however no such effect has ever been reported *in vivo*. The presence of physiological concentrations of the V2R-specific analogue [deamino-Cys1,D-Arg8]-vasopressin (dDAVP) decreased caspase-3 and -7 cleavage in renal tubular cells [14], while the effect of vasopressin against serum deprivation-induced apoptosis on neuronal cell line H32 was exerted through protein kinase C α/β and ERK/ MAPK-mediated phosphorylation (inactivation) of the pro-apoptotic protein Bad, as well as activation of the phosphoinositide 3 kinase (PI3K)/Akt pathway [15]. Our data present the first set of evidence that activation of hepatocyte-specific V1R by lysine vasopressin effectively activates the canonical Wnt signaling and exerts potent hepatoprotective effects *in vivo* in a clinically relevant mouse model. However the Wnt/β-catenin/FoxO3a/AKT pathway is unlikely to be the solely important signaling pathway downstream of V1R. In support of this notion, a recent study using quantitative phosphoproteomics with iTRAQ labeling has revealed activation of multiple signaling pathways other than the canonical GPCR-activated calcium signaling upon V2R activation in renal collecting ductal epithelium, including the PI3K, P38 MAPK, VEGF, Wnt/β-catenin pathways, etc [14].

Another novel finding in the current study is that hepatic IR induces AVP secretion and hepatocyte V1R expression. AVP is a peptide hormone which is formed in the hypothalamus and released from the posterior pituitary. Its release to peripheral blood can be triggered by hypovolemia, hypotension, increase osmolarity, increased angiotensin II, etc [16]. Although it remains elusive as to how IR triggers the release of AVP, we believe this is most likely due to the transient hypotension caused by decreased venous blood return to the heart after the total portal pedicle occlusion, as well as reperfusion-induced hypotension [17]. Besides, other mechanisms may operate in this setting. Several studies have demonstrated that peripheral osmoreceptors modulated AVP release from the posteriorpituitary into the circulation, and these osmoreceptors are located within the liver and portal vein region. Another possible explanation is that hepatic IR may induce the release of gastrointestinal hormones or ouabain-like substances, which activate the hypothalamic nuclei leading to AVP secretion [18, 19]. The detailed mechanism needs to be investigated in future studies. On the other hand, liver IR up-regulates V1R expression in hepatocytes. Very few studies have shed light on the regulation of V1R expression [2]. Glucocorticoids was able to enhance V1R expression via increasing mRNA stability in vascular smooth muscle cells [20], cyclosporine A and dexamethasone also increased V1R expression in proliferating skeletal muscle cells [21], while the expression of V1R was down-regulated during sepsis [22]. We have no clear hypothesis regarding the molecular mechanism of induced V1R expression at this stage, however significant amount of *in vitro* and *in vivo* results from our study support the functional importance of the hepatocyte-specific V1R expression upon IR. Although difference may exist between the rodent and human, nonetheless, given the expression of V1R in human livers [23], the similar pathogenesis of hepatic IRI between human and rodent animals, and the beneficial effect of using terlipressin in patients who already have compromised liver function, we strongly believe this AVP-V1R axis may also plays a hepatoprotective role in the clinical scenario.

Although we used a clinically relevant model in the current study, and we did provide some preliminary data regarding the benefit of using terlipressin in patients with ESLD, one major caveat is the lack of direct clinical evidence to support the rational of using terlipressin in patients who suffered from liver IRI. Currently we are conducting a clinical prospective trial to prove the hepatoprotective effect of using terlipressin in patients who require inflow control during hepatectomy, and to determine the optimal timing and dosage of terlipressin administration.

In summary, we provide solid evidence that terlipressin treatment has potent hepatoprotective effects both *in vitro* and *in vivo*. Our findings not only have immediate clinical significance which may help to alleviate the concerns of using terlipressin in patients with ESLD who already have compromised hepatic function, but can be translated into therapeutic use for liver protection in many pathological circumstances such as inflow occlusion during hepatectomy, liver transplantation, liver steatosis and small-for-size livers.

## MATERIALS AND METHODS

### Patients

We conducted a prospective non-randomized observational study. Nineteen patients with advanced liver cirrhosis and ascites who had received terlipressin treatment for various reasons were enrolled. The diagnosis of cirrhosis was documented by liver biopsy or the presence of chronic liver diseases shown by hepatic insufficiency and/or signs of portal hypertension combined with hematological, ultrasonograghic and endoscopic findings. Among these patients, 14 had a chronic hepatitis B infection and 2 had chronic hepatitis C infection. 3 patients had a hepatoma, 2 of them also had chronic hepatitis B infection. One patient with alcoholic cirrhosis and one patient with schistosomal cirrhosis were also included. Patients were excluded if they had gastrointestinal bleeding within two weeks prior to the study, encephalopathy, previous surgical portocaval shunt, unstable co-morbidities such as infections or lung diseases, general anesthesia within one month, clinically significant cardiovascular diseases, or known history of drug and alcohol abuse within two months. Furthermore, albumin infusion was temporarily discontinued 72 hours before the investigation. Terlipressin was administered as an intravenous bolus at a dose of 1mg at 12-h intervals for a total of 3-7 days, liver function tests were performed before terlipressin treatment (day 0) and 4 days after completion of therapy.

### Animals

Male wild-type C57BL/6 mice were purchased from the Shanghai Laboratory Animal Center (Chinese Academy of Sciences, Shanghai, China) and used at 8-10 weeks of age. The animals were housed under specific pathogen-free conditions and received humane care according to the criteria outlined in the National Guideline for the Care and Use of Laboratory Animals. The animal protocol was approved by the Institutional Animal Care and Use Committee of ZJPPH.

### Reagents

A commercially available synthetic 12-peptide terlipressin (3′glycyl lysine vasopressin, HyBio Pharmaceutical Ltd, Shenzhen, PR China) was used for mouse therapy. Lysine vasopressin (Lypressin) was synthesized and kindly provided by Dr. Jian Liu from the R&D department of HyBio Pharmaceutical Ltd. Both drugs were freshly prepared immediately before use according to the manufacturer's instructions. The following antibodies were used in the western blot: V1R (1:200, Santa Cruz Biotechnology, Inc., Santa Cruz, CA), cleaved-caspase-3, B-cell lymphoma 2 (BCL-2), B-cell lymphoma-extra large (BCL-XL), β-catenin, forkhead box O3a (FoxO3a), phospho-forkhead box O3a (P-FoxO3a), Bcl-2 interacting mediator of cell death (Bim), P27, Wnt3a, phospho-protein kinase B (P-AKT), AKT and glyceraldehyde-3-phosphate dehydrogenase (GAPDH) (1:1000, Cell Signaling Technology, Inc., Danvers, MA). The following small molecule compounds were used *in vitro* as specific pathway antagonists: V1R inhibitor SR49059 (Sigma-Aldrich, St. Louis, MO) and Wnt/beta-catenin inhibitor XAV-939a (Selleck, Houston, TX). The following reagents were used *in vitro*. 2- [4-(2-Hydroxyethyl)-1-piperazinyl] ethanesulfonic acid (HEPES), potassium chloride (Kcl), glucose, Ethylenediaminetetraaceticacid (EDTA), phenol red solution, calcium chloride (CaCl2) and sodium hydroxide (NaOH) were purchased from Sigma (St Louis, MO, USA). Collagenase H was bought from Roche (Roche, Indianapolis, IN). Williams E medium and fetal bovine serum was obtained from Gibco (Gibco, Gaithersburg, MD, USA).

### Liver IR model and treatment

We adopted a warm partial mouse hepatic ischemia-reperfusion injury (IRI) model in which 70% of the liver mass underwent 90 minutes (min) of warm ischemia and various durations of reperfusion, as described previously [24]. Sham-operated controls underwent the same procedure but without vascular occlusion. The IR group received a single-dose administration of terlipressin (0.1 mg/kg) or the same volume of normal saline via the dorsal penile vein either 10 min prior to occlusion of the arterial and portal venous blood supplying the cephalad lobes (before group), or immediately after lobetic reperfusion (after group). We did try earlier time points of terlipressin administration (30 or 60 min before lobetic ischemia), however neither had yielded better results than the time chosen in this experiment (data not shown). Mice were sacrificed at different time points after reperfusion for sample collection.

### Assessment of liver injury

Alanine transaminase (ALT), aspartate aminotransferase (AST), total bilirubin as well as lactate dehydrogenase (LDH) levels were measured using commercially available kits (Abnova, Taiwan) according to the manufacturer's instructions.

### Isolation and culture of hepatocytes

Hepatocytes were isolated from normal C57BL/6 (B6) mice as described previously [25]. In brief, the liver was cannulated via the portal vein and perfused with perfusion solution (1×PBS with 10mM HEPES, 0.05% Kcl, 5mM glucose, 200uM EDTA and 0.001% phenol red solution, PH 7.4) until the liver became clear of blood. When the liver began to get congested, it was continuously perfused with digestive medium (0.05% Kcl, 1×PBS with 30mM HEPES, 1mM CaCl2, 5mM glucose, 0.2% phenol red solution and 0.02% Collagenase H, PH 7.4). After perfusion was completed, the liver was dissected out and fractionized in the presence of Williams E medium with 10% fetal bovine serum. Non-parenchymal cells (NPCs) were separated by three cycle of differential centrifugation at 400 rpm for 10 minutes. Hepatocyte suspensions were then separated by 40% percoll centrifugation at 400 rpm for 10 minutes. The cells were washed twice and cultured for additional 24 hours in Williams E medium with 10% fetal bovine serum before treatments.

For experiments involving hypoxia/ reoxygenation (H/R), hepatocyte growth medium equilibrated with 3% O2/92% N2/5% CO2 or 95% O2/5% CO2 were used as hypoxia or reoxygenation medium, respectively. In the hypoxia group, after pretreatment with lysine vasopressin, cells were cultured in 2 ml hypoxia medium at 37°C in an airtight chamber flushed with the same gas mixture. In the reoxygenation group, the culture medium was replaced with 2 ml reoxygenation medium in the presence of lysine vasopressin, and cells were further incubated at 37°C in an airtight chamber flushed with the same gas mixture. To study the hepatoprotective effects of lysine vasopressin, primary mouse hepatocytes were cultured in the presence or absence of 0.33 or 1.0 nM of lysine vasopressin, and stimulated with 4 hours of hypoxia (hypoxia group), or 4 hours of hypoxia followed by 1 or 4 hour of reoxygenation (reper 1 and 4 hour group, respectively). To testify the functional significance of the V1R- Wnt/β-catenin pathway, mouse primary hepatocytes were pretreated with SR49059 (10 uM) for 10 min or XAV-939a (1uM) for 24 hours, followed by hypoxia and 4 hours of reoxygenation in the presence or absence of lysine vasopressin (1.0 nM).

### Knockdown of V1R in NCTC cells

NCTC cells (immortal mouse hepatocyte cell lines) were purchased from American Type Culture Collection (ATCC). NCTC cells with deficiency of V1R expression were established with lentiviral transfection of V1R-specific short hairpin RNA (shRNA). The sequences of V1R-shRNA were as follows: V1R/ Knockdown 1 (KD1): 5′ -TTTGAGGTGAACAATGGCA-3′, V1R/KD2: 5′ -TTTCGTTTGGACCGATTCC-3′, V1R/KD3: 5′ -AGCATAGCGCAGAAATTCG-3′, V1R/ negative control (NC): 5′-GCCTATCGCTGTTCTTGAA-3′. KD3 sequence was chosen to knock down V1R due to its highest efficiency (data not shown).

### Quantitative reverse transcription polymerase chain reaction (RT-PCR)

Quantitative RT-PCR was performed using the DNA Engine with the Chromo 4 detector (MJ Research, Waltham, MA). Target gene expression was calculated relative to the housekeeping gene, hypoxanthine-guanine phosphoribosyl transferase (HPRT). The sequence of primer sets used for target genes were listed in Supplementary Table 2.

### Western blot

Equal amounts of protein samples were separated by sodium dodecyl sulfate polyacrylamide gel electrophoresis (SDS-PAGE) on an 8-15% bis-tris gel and electrophoretically transferred onto 0.2μm polyvinylidene fluoride membrane (Merck Millipore, Darmstadt, Germany). Membranes were blocked with 5% non-fat milk and sequentially incubated with primary and horseradish peroxidase (HRP)-conjugated secondary antibodies. Bound antibodies were visualized using enhanced chemiluminescent substrate (ECL; Thermo Scientific, Waltham, MA). Relative quantities of protein were determined by densitometry using Quantity One® (Bio-Rad).

### Enzyme-linked immunosorbent assay (ELISA)

Levels of monocyte chemotactic protein 1 (MCP-1), interleukin-6 (IL-6) and tumor necrosis factor-α (TNF-α) in the supernatant were measured using mouse ELISA kits (eBioscience, San Diego, CA) according to the protocol provided by the manufacturer. Serum levels of AVP were measured using the AVP ELISA kit (Cayman Chemical Company, Ann Arbor, MI) following the manufacturer's instructions. All experiments were performed three times in triplicate.

### Histology and immunohistochemistry

Formalin-fixed liver tissues were stained with hematoxylin and eosin (H&E) to evaluate necrosis and inflammatory infiltration. For apoptosis detection, tissue sections were stained with terminal deoxynucleotidyl transferase (TdT)-mediated dUTP digoxigenin nick-end labeling (TUNEL) according to the manufacturer's instructions (In situ cell death detection kit, POD, Roche). For immunohistochemical staining of the infiltrating immune cells, primary monoclonal antibody (mAbs) against mouse Ly-6G (1A8; BD Biosciences, San Jose, CA) and CD68 (FA-11; AbD Serotec, Raleigh, NC) were used on liver specimens. Positive cells were counted in 10 high-power fields (HPF)/section (×400).

### Myeloperoxidase (MPO) assay

The hepatic MPO activity was measured using myeloperoxidase peroxidation fluorometric assay kit (Cayman Chemical Company, Ann Arbor, MI) according to the protocol provided by the manufacturer. One absorbance unit (U) of MPO activity was defined as the quantity of enzyme degrading 1 mol peroxide/min at 25°C/gram of tissue.

### Hepatic hemodynamic study

All examinations were performed on a 64-row MDCT system (Discovery CT750 HD; GE Healthcare, Milwaukee, WI). Mice were scanned cranio-caudally in supine position. Unenhanced scans were acquired following scout scans with helical scan mode at 80kVp tube voltage, 80 mA, and rotation time 0.5s. Mice were then injected with nonionic contrast medium (Iopamidol Injection, Iopamiro 370; Shanghai BRACCO Sine Pharmaceutical Corp. Ltd., China) via the inferior vena cava (IVC) at a rate of 0.1ml/sec for a total of 0.2ml during the perfusion scanning. The scanning delay for perfusion imaging was about 5 seconds after the injection, and the scanning duration was 50 seconds. Perfusion scans were performed using cine mode (axial) with 80kVp and cine time 0.5s, scan interval 1.5s. Other scanning parameters were as follows: collimation width: 0.625mm*64, scanning coverage: 1.25mm*32, SFOV: 25cm, tube current: 80mA, rotation speed: 0.5s. Images were reconstructed with a standard recon kernel. All images were reconstructed with 1.25mm slice thickness. The axial images with time series were loaded on an advanced workstation (AW4.5; GE Healthcare, Waukesha, Wisconsin USA) with the software CT perfusion 4 (GE Healthcare, Waukesha, Wisconsin). Free hand ROIs (region of interest) were drawn to encompass as much of the hepatic parenchyma as possible excluding the vessels. Quantitative parameters of blood flow were obtained from the functional maps. Each measurement was performed on three adjacent images and the mean value of the three measurements was recorded.

### Statistics

Statistical analysis was performed using the Student t test or one-way analysis of variance (ANOVA). All statistical analyses were performed and graphs were generated using GraphPad Prism 5. The *p* value < 0.05 was considered statistically significant.

## SUPPLEMENTARY MATERIAL FIGURES AND TABLES


