# Supplementary material for: Signaling through hepatocyte vasopressin receptor 1 protects mouse liver from ischemia-reperfusion injury
Source: Oncotarget. 2016 Oct 4;7(43):69276–90. doi: 10.18632/oncotarget.12472 (PMC5342477; doi:10.18632/oncotarget.12472)
Supplement: Supplementary file 1 [file oncotarget-07-69276-s001.pdf]

Signaling through hepatocyte vasopressin receptor 1 protects mouse liver from ischemia-reperfusion injury

Supplementary Material

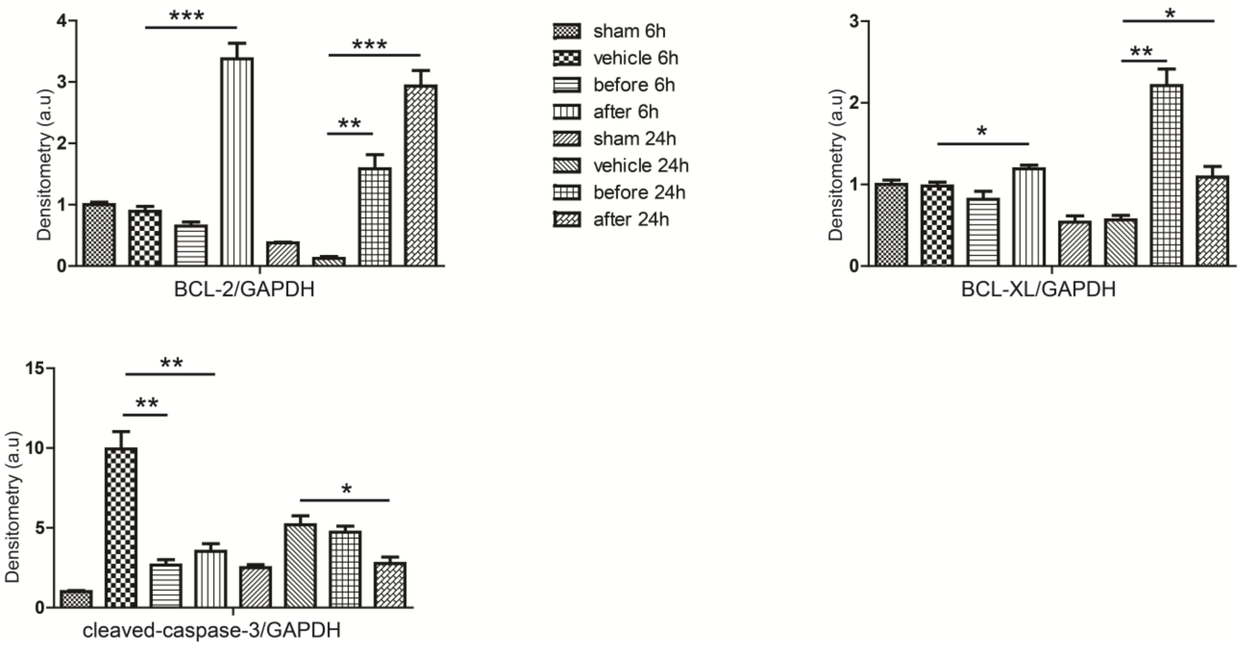

Fig. S1. Densitometric analyses of western blot (Fig. 4C) for BCL-2, BCL-XL and cleaved-caspase-3 expressions, respectively. Values shown were relative to sham group after 6 hours of reperfusion (set as 1). *$p<0.05$; **$p<0.01$; ***$p<0.001$.

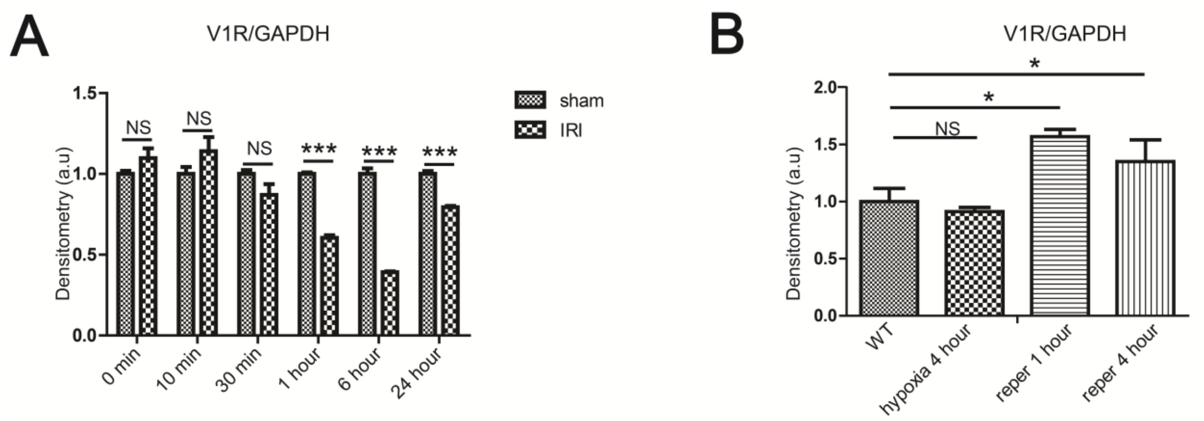

Fig. S2. (A) Densitometric analyses of western blot (Fig. 5B) for V1R expression. Values shown were relative to sham group after 0 minutes of reperfusion (set as 1). NS, no significance. (B) Densitometric analyses of western blot (Fig. 5C) for V1R expression. Values shown were relative to WT group (set as 1). NS, no significance; *p<0.05; ***p<0.001.

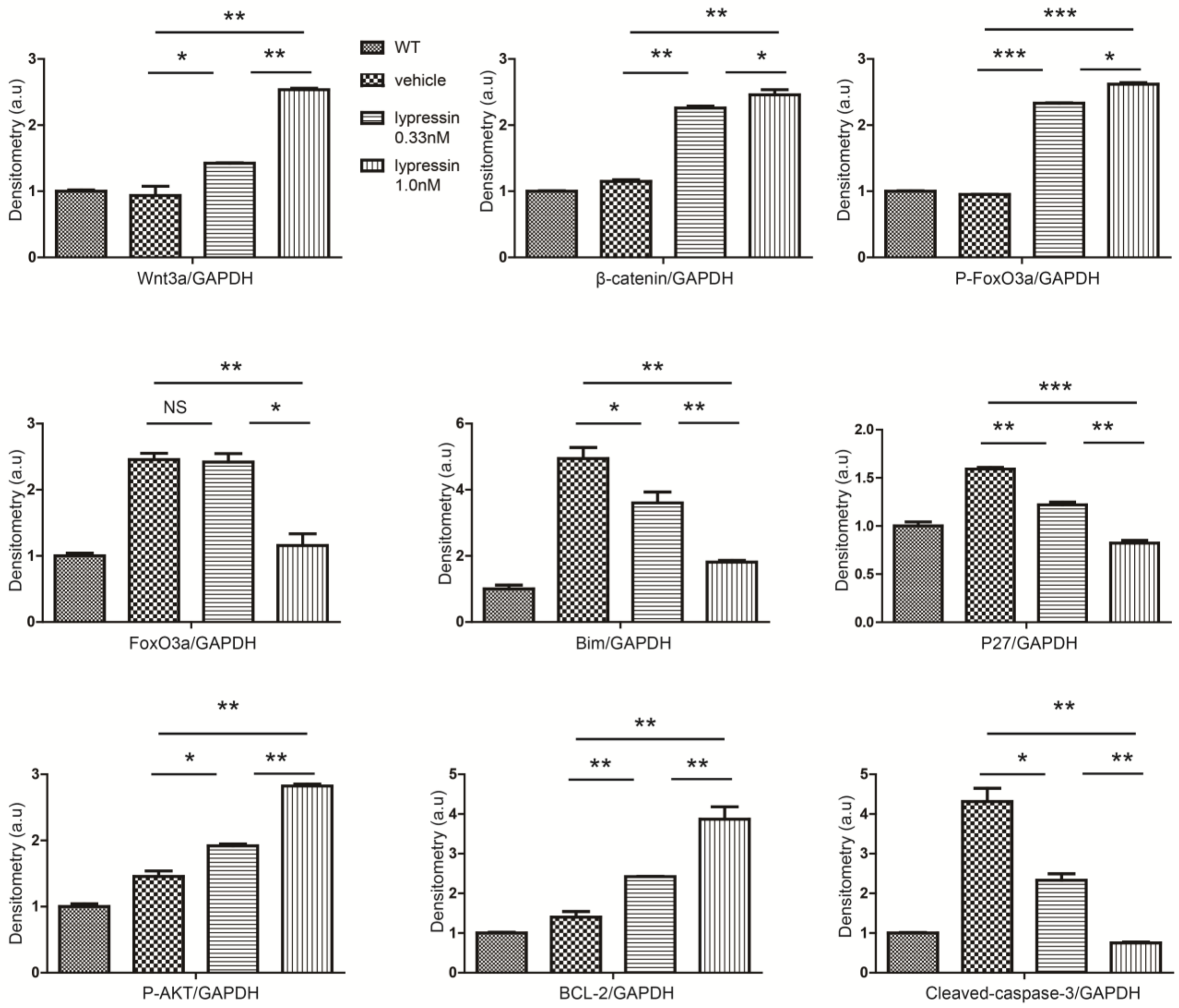

Fig. S3. Densitometric analyses of western blot (Fig. 7A) for Wnt3a, β-catenin, Phospho-FoxO3a, FoxO3a, Bim, P27, Phospho-AKT, BCL-2 and cleaved-caspase-3, respectively. Values shown were relative to vehicle group (set as 1). *p<0.05; **p<0.01; ***p<0.001, NS, no significance.

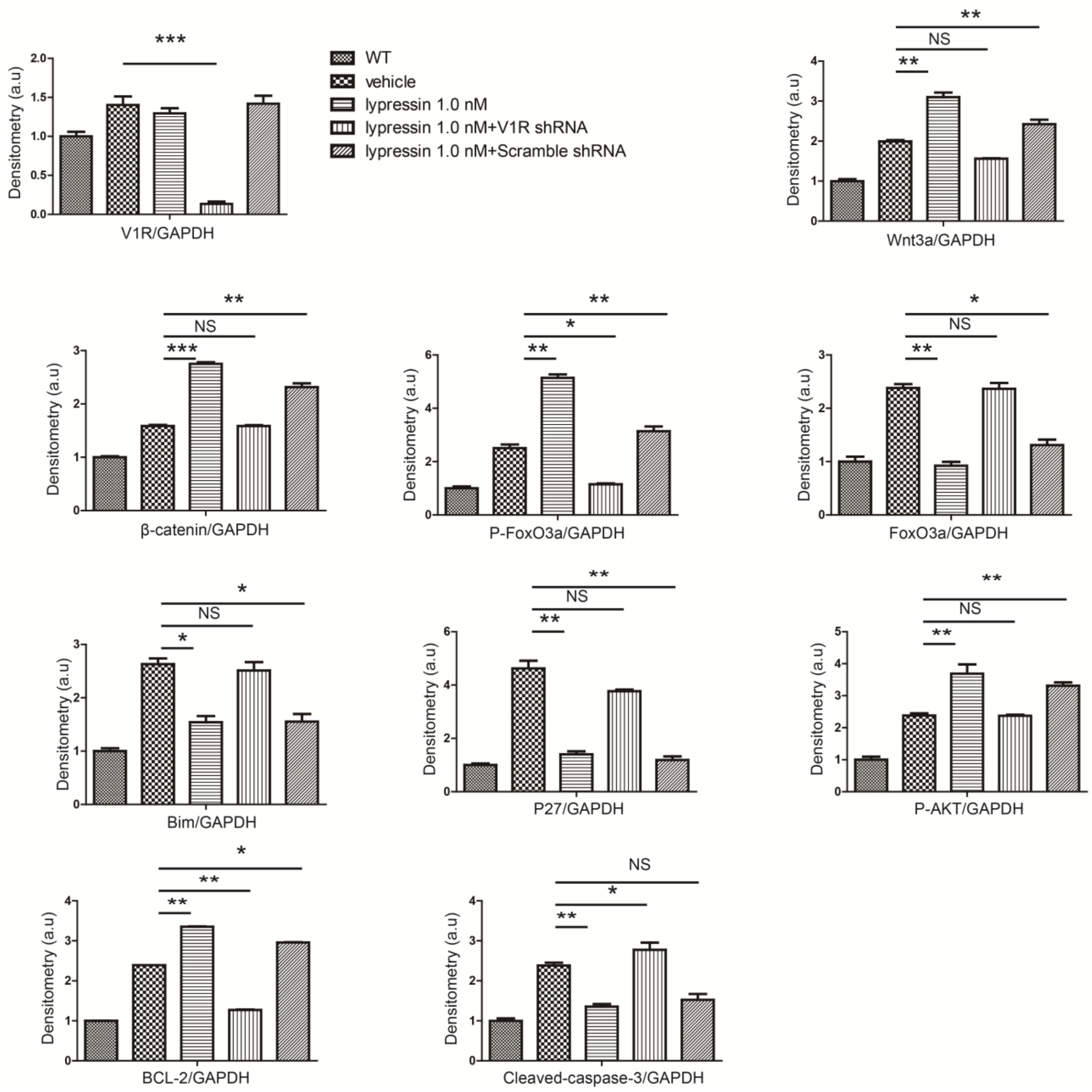

Fig. S4. Densitometric analyses of western blot (Fig. 7B) for V1R, Wnt3a, β-catenin, Phospho-FoxO3a, FoxO3a, Bim, P27, Phospho-AKT, BCL-2 and cleaved-caspase-3, respectively. Values shown were relative to vehicle group (set as 1). *p<0.05; **p<0.01; ***p<0.001, NS, no significance.

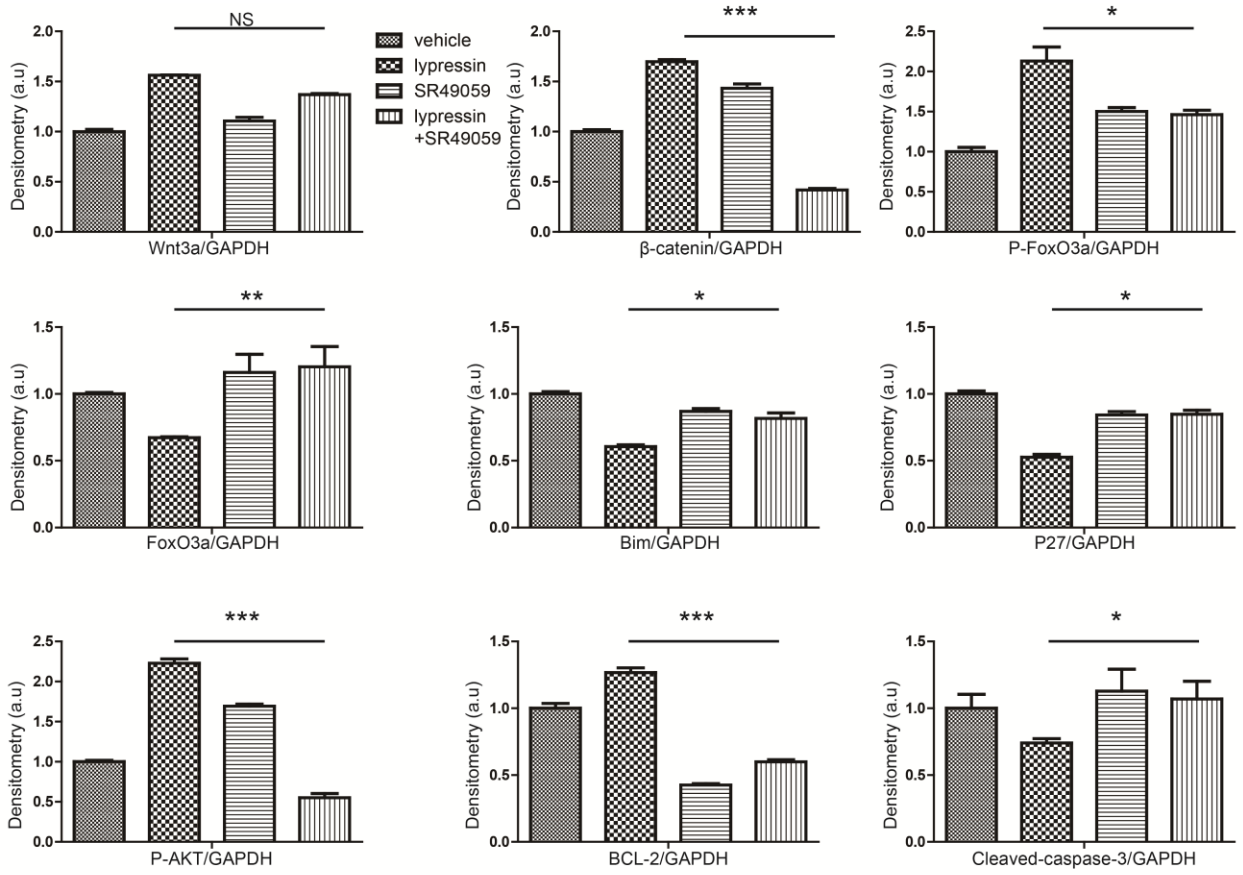

Fig. S5. Densitometric analyses of western blot (Fig. 7C) for Wnt3a, β-catenin, Phospho-FoxO3a, FoxO3a, Bim, P27, Phospho-AKT, BCL-2 and cleaved-caspase-3, respectively. Values shown were relative to vehicle group (set as 1). *p<0.05; **p<0.01; ***p<0.001, NS, no significance.

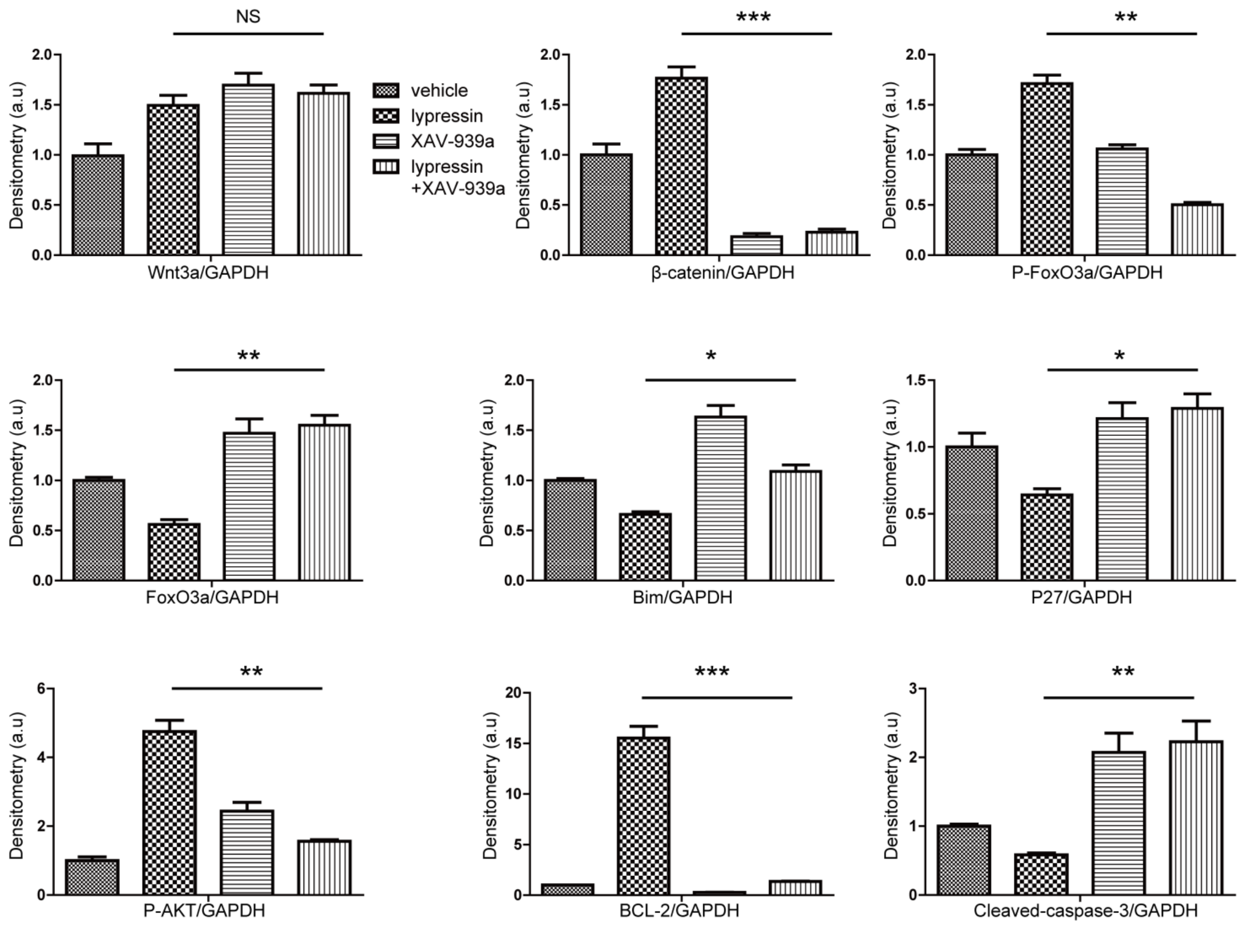

Fig. 6. Densitometric analyses of western blot (Fig. 7D) for Wnt3a, β-catenin, Phospho-FoxO3a, FoxO3a, Bim, P27, Phospho-AKT, BCL-2 and cleaved-caspase-3, respectively. Values shown were relative to vehicle group (set as 1). *p<0.05; **p<0.01; ***p<0.001, NS, no significance.

Supplementary Table 1. Demographic data of patients with ESLDs.

Characteristic	Value	Range
Ages (years)	56.53±13.77	34-76
Sex		
Male	14	
Female	5	
Etiology		
Hepatitis	16	
Hepatoma	3	
others	2	
TBIL (umol/L)	386.70±298.90	12.6-820.60
ALB (g/L)	30.72±5.45	20.8-44.8
ALT (U/L)	74.84±53.54	15-195
AST (U/L)	138.60±98.93	23-396
CPT Score	9.94±1.22	8-12

*Values were provided in mean ±SD. ESLDs, end-stage liver diseases; TBIL, total bilirubin; ALB, albumin; ALT, alanine transaminase; AST, aspartate aminotransferase; CPT score, Child-Pugh-Turcotte score.

Supplementary Table 2: Sequence of primer pairs used in real-time quantitative PCR.

Genes	Forward primer	Reverse primer
IL-6	CCAATGCTCTCCTAACAGA	TGTCCACAAACTGATATGC
TNF-α	CTCTTCAAGGGACAAGGCTG	CTCTTCAAGGGACAAGGCTG
IL-1β	TTCAGGCAGGCAGTATCA	GTCACACACCAGCAGGTTA
MCP-1	AAAACACGGGACGAGAAACCC	ACGGGAACCTTTATTAACCCCT
V1R	TTCTTCACATTTCCCATCC	TTCTGTCTGTCTCCCGTTT
IFN-γ	CAG CAA CAA CAT AAG CGT C	CTC AAA CTT GGC AAT ACT C
HPRT	TCAACGGGGGACATAAAAGT	TGCATTGTTTTACCAGTGTCAA
